# The relationship between thyroid peroxidase antibody and differentiated thyroid cancer: a systematic review and meta-analysis

**DOI:** 10.3389/fendo.2024.1349041

**Published:** 2024-02-27

**Authors:** Haonan Zhang, Lijun Tian, Xichang Wang, Xiaoguang Shi

**Affiliations:** ^1^ Department of Endocrinology and Metabolism, Shengjing Hospital of China Medical University, Shenyang, Liaoning, China; ^2^ Department of Endocrinology and Metabolism, Institute of Endocrinology, The First Affiliated Hospital of China Medical University, Shenyang, Liaoning, China

**Keywords:** differentiated thyroid cancer, thyroid peroxidase antibody, prevalence, prognosis, meta analysis

## Abstract

**Background:**

Thyroglobulin antibody (TgAb) has been found to be associated with the occurrence and development of differentiated thyroid cancer (DTC) for several years, but there is still controversy over whether thyroid peroxidase antibody (TPOAb) is related to differentiated thyroid cancer.

**Methods:**

We scrutinized relevant studies published up to July 2023 across four major databases including PubMed, Embase, Cochrane Library, and Web of Science, to examine the association between TPOAb and DTC. Clinical outcome measures include the incidence of DTC, tumor size, extrathyroidal invasion, lymph node metastasis, multifocality, recurrence and bilaterality.

**Results:**

12 original studies were included, involving a total of 20,330 subjects. Our analysis of the included studies revealed that TPOAb+ individuals exhibited a higher risk of developing DTC (OR=1.57 [95% CI: 1.00–2.45], p=0.049) than TPOAb– individuals. Furthermore, TPOAb+ DTC patients were more prone to present with bilateral (OR=1.40 [95% CI: 1.21–1.62], p<0.00001) and multifocal (OR=1.40 [95% CI: 1.23-1.60], p<0.00001) tumors than TPOAb– patients. Sensitivity analysis indicated a high sensitivity for these three findings. No significant differences in the risk of extrathyroidal extension and lymph node metastasis, recurrence rate, tumor size, were observed between TPOAb+ and TPOAb– DTC patients.

**Conclusion:**

The presence of TPOAb is correlated with an increase prevalence of DTC. However, its effectiveness as a prognostic marker for DTC patients warrants further investigation.

**Systematic review registration:**

https://www.crd.york.ac.uk/PROSPERO/, identifier CRD42023448824.

## Introduction

Thyroid cancer is one of the most prevalent endocrine malignancies, with its annual incidence increasing in numerous countries and regions. However, the mortality rate remains consistently low (1, 2). Differentiated thyroid cancer (DTC) is the most frequently diagnosed type, accounting for over 95% of cases (3). DTC comprises two main subtypes, with papillary thyroid cancer (PTC) account for 90% of cases and follicular thyroid cancer accounting for 7–8% (4–6). In recent years, increasing studies have reported the association of thyroid cancer with various risk factors, including radiation exposure, environmental factors, iodine intake, serum TSH levels and Hashimoto’s thyroiditis (HT) (7–9). Among these factors, thyroid autoimmunity is closely linked to the development of DTC.

Thyroid peroxidase (TPO), an enzyme located in the apical membrane of thyroid follicular cells, is involved in the biosynthesis of thyroid hormone (10). Thyroid peroxidase antibody (TPOAb) is predominantly produced by lymphocytes infiltrating the thyroid gland and is one of the most common autoantibodies against the thyroid (11). It can cause thyroid cell damage through the activation of the complement system and cell cytotoxicity (12, 13). TPOAb is a hallmark thyroid autoimmune antibody in autoimmune thyroiditis (AIT) (14, 15). It is also intricately linked to several other diseases. In 1998, Smyth et al. observed significantly higher TPOAb positivity in breast cancer patients than in those with benign breast disease. Some studies have shown that TPO is expressed in breast cancer and peri-cancerous tissues, and the antigenic and biochemical properties of TPO in breast tissues are similar to those in thyroid tissues, with only minor differences in post-translational modifications (16–18). Furthermore, disease-free and overall survival were longer in TPOAb+ breast cancer patients than in their TPOAb– breast cancer counterparts (19). Numerous subsequent studies have yield similar findings (20–22). Sharma and collaborators proposed that TPOAb might cross-react with lactoperoxidase expressed in breast tissue, potentially explaining the coexistence of TPOAb and breast cancer (23). Additionally, several studies have highlighted a strong association between TPOAb and various adverse pregnancy outcomes (24–26). Furthermore, two separate fine needle aspiration cytology studies from the same center identified TPOAb as an independent risk factor for thyroid malignancy (27, 28). Nonetheless, while many studies have explored the relationship between thyroglobulin antibody (TgAb), another serum marker of AIT, and DTC, the association between TPOAb and DTC remains unclear (29–31).

Previously, a variety of diseases or molecules have been found to be associated with the prognosis of DTC, such as graves’ disease, HT, thyrotropin, thyroglobulin (Tg), BRAF mutation, estrogen receptor and VEGF pathway, etc (32–38). And we aimed to conduct a comprehensive meta-analysis and systematic review to elucidate the relationship between TPOAb and DTC. By analyzing extensive large-scale research data, we expect to provide highly in-depth theoretical support for the clinical application of TPOAb in the early diagnosis, formulation of treatment strategies, and prognosis evaluation of DTC.

## Methods

### Registration

This systematic review was registered (CRD42023448824) in PROSPERO. We continue to update this systematic review with the latest information.

### Search strategy

Our systematic literature search encompassed four databases: PubMed, Embase, Web of Science, and Cochrane Library, until July 2023. The search was conducted using the following terms: “thyroid peroxidase antibody” AND “papillary thyroid cancer” OR “follicular thyroid cancer” OR “Hürthle cell thyroid cancer” OR ‘‘differentiated thyroid cancer.” **Supplementary Table S1** provides a comprehensive outline of the search strategy. We selected relevant articles through the evaluation of titles, abstracts, and full texts. Two researchers independently made the selections and reviewed the abstracts and full texts. In instances where multiple original studies involving the same population were published, we selected the most recent and comprehensive study.

### Inclusion and exclusion criteria

Studies that met the following criteria were included (1): prospective, retrospective, randomized controlled trial, or case-control study types (2), investigation of TPOAb levels in patients diagnosed with DTC (3), classification of patients based on their TPOAb levels (4), availability of relevant data, and (5) publication in English.

Studies that meet any of the following criteria were excluded (1): review articles, letters, comments, editorials, case reports, or laboratory-animal research (2), incomplete data or unavailability of raw data, or (3) duplication of studies originating from the same dataset.

Two researchers independently screened articles, with any disputes resolved through negotiation or with the assistance of a third author.

### Data extraction

We extracted the following information from each included article (1): last name of first author (2), publication year (3), study period (4), country (5), study type (6), sample size (7), total number of TPOAb± patients (8), mean tumor sizes in TPOAb± groups (9), lymph node metastasis in TPOAb± groups (10), extrathyroidal extension in TPOAb± groups (11), tumor multifocality in TPOAb± groups (12), tumor bilaterality in TPOAb± groups, and (13) cancer recurrence in TPOAb± groups.

### Quality assessment

In this meta-analysis, two reviewers used the Newcastle Ottawa quality assessment scale (NOS) to evaluate the quality of the included studies. The NOS assesses the selection of study subjects, comparability between groups, and measurement or exposure of outcomes. Each study received a score ranging from 0 to 9 points. Studies with scores exceeding 6 were considered high quality, those with scores between 4 and 6 were considered moderate quality, and those with scores below 4 were considered low quality. Any discrepancies or inconsistencies were resolved through consensus with a third author.

### Statistical analyses

We conducted statistical analyses using Review Manager 5.4 (Copenhagen: The Nordic Cochrane Centre. The Cochrane Collaboration) and Stata 14 (StataCorp LP, College Station, Texas). The forest plots were generated using Review Manager 5.4. Odds ratio (OR) with a 95% confidence interval (Cl) was used to assess the strength of the association between TPOAb and DTC. Weighted mean difference and 95% Cl were calculated for continuous outcomes. In all meta-analyses, the Cochrane Q p-value and l² statistic were used to assess heterogeneity. A random-effect model was employed to merge results when the p-value was < 0.05 or I² > 50%, indicating significant heterogeneity; otherwise, a fixed-effect model was used. Statistical significance was set at p < 0.05 was considered statistically significant. Publication bias was assessed using Egger test plots in Stata 14.

## Results

### Study selection

**Figure 1:**
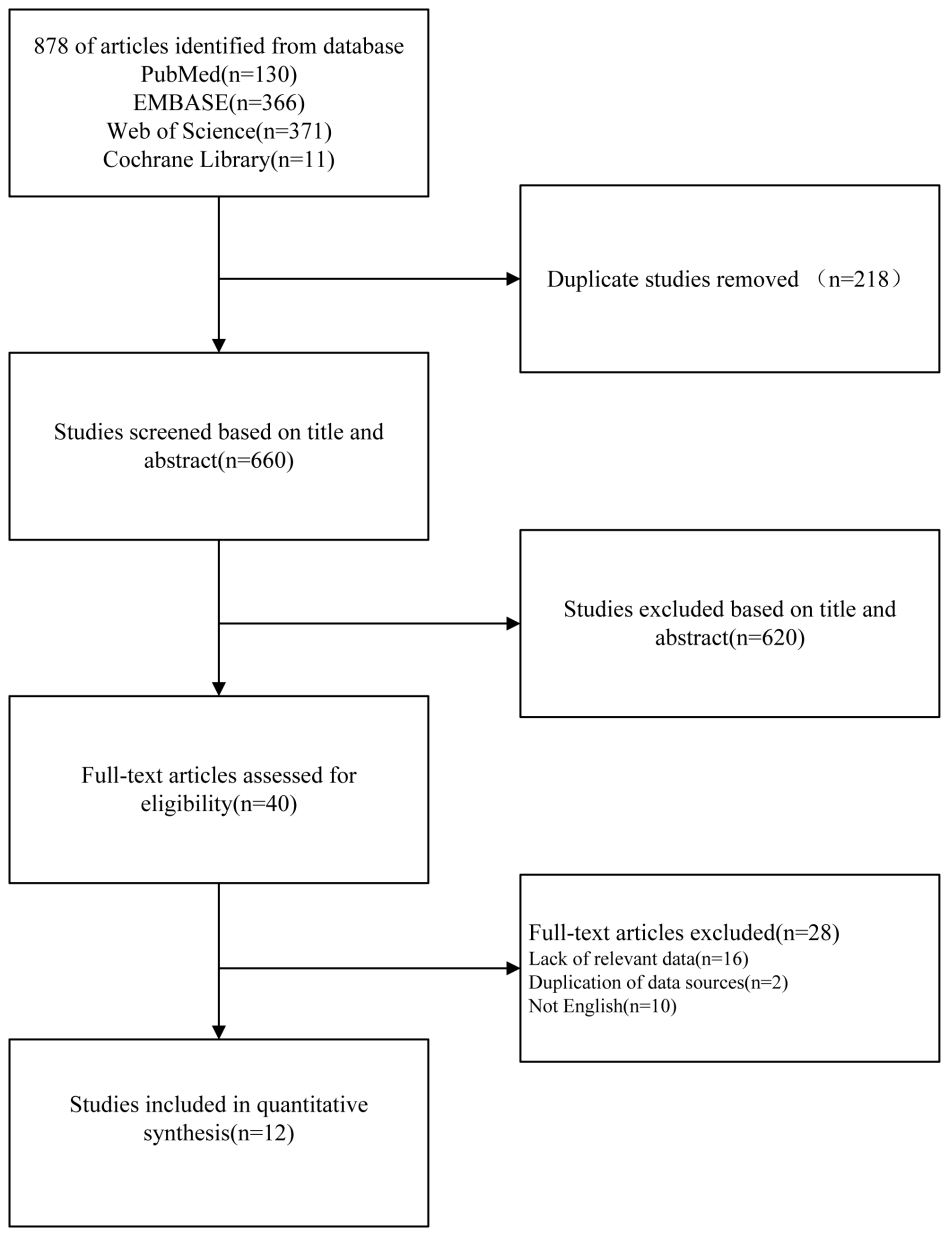
Flowchart of the inclusion or exclusion procedure.

We conducted a comprehensive literature search using PubMed, Embase, Cochrane Library, and Web of Science databases as well as by examining references in relevant reviews up to July 2023. Initially, we identified 878 records. After removing 218 duplicated articles, we screened titles and abstracts, resulting in the selection of 40 studies out of 660 articles. Following a full-text review and application of inclusion and exclusion criteria, we included 12 original studies in the systematic review and meta-analysis. A visual representation of this selection process, following the PRISMA guidelines, is provided in **Figure 1**.

### Characteristics of included studies

The key characteristics of the 12 eligible studies are summarized in **Table 1** (39–50). These 12 case-control studies encompassed 20,330 participants across the United States, China, Greece, Serbia, Korea, and Turkey. We categorized nine studies as having moderate quality and three as high quality. The sample size of the included studies ranged from 179 to 5770. We explored DTC prevalence, tumor size, extrathyroidal extension, lymph node metastasis, tumor multifocality, tumor bilaterality, and cancer recurrence between TPOAb+ and TPOAb– patients across these studies.

**Table 1:** Main characteristics of all studies included in the meta-analysis.

Study	Publication year	Study type	Study period	Country	Sample size	TPOAb+ threshold	TPOAb+ individuals	TPOAb– individuals	TPOAb detection time	Outcome	NOS score
Paparodis	2023	Retrospective	2000-2013(United States) 2007-2021(Greece)	United States、Greece	1635	>34IU/mL	540	1095	Within 3 months before surgery	DTC incidence, tumor size, ETE, LNM	7
Wang	2022	Retrospective	2012-2016	China	5770	>100IU/mL	1123	4647	1 to 2 days before surgery	ETE, MF, LNM, recur, BF	6
Chen	2022	Retrospective	2014-2020	China	319	>34IU/mL	52	267	Before surgery	DTC incidence	7
Huang	2022	Retrospective	2000-2021	China	179	>9IU/mL	64	115	Before surgery	ETE, MF, LNM, recur, BF	6
Li	2021	Retrospective	2015-2018	China	3934	>5.61IU/mL	815	3119	Before surgery	DTC incidence	6
Zorić	2020	Retrospective	2008-2012	Serbia	263	N/A	33	95	NA	DTC incidence	8
Peng	2020	Retrospective	2018-2019	China	322	≥9IU/mL	134	188	Before surgery	DTC incidence	6
Song	2019	Retrospective	2007-2011	China	2070	>60IU/mL	389	1681	Within 90 days before surgery	ETE, LNM,	6
Selek	2017	Retrospective	2009-2014	Turkey	870	>9 IU/mL	228	642	Before surgery	DTC incidence	6
Zeng	2016	Retrospective	2003-2012	China	1198	>20IU/mL	121	870	Before surgery	DTC incidence	6
Qin	2015	Retrospective	2011-2013	China	1638	>5.61IU/mL	136	1502	Before surgery	DTC incidence, tumor size, ETE, LNM, MF	6
Wu	2014	Retrospective	2006-2011	China	2132	>5.61IU/mL	82	2050	NA	DTC incidence, tumor size, ETE, LNM, MF	6

TPOAb+, thyroid peroxidase antibody positive; TPOAb–, thyroid peroxidase antibody negative; DTC, differentiated thyroid cancer; ETE, extrathyroidal extension; LNM, lymph node metastasis; MF, tumor multifocality; BF, tumor bilaterality; recur, cancer recurrence; NOS, Newcastle–Ottawa scale; N/A, not applicable.

### TPOAb positivity and DTC prevalence

**Figure 2:**
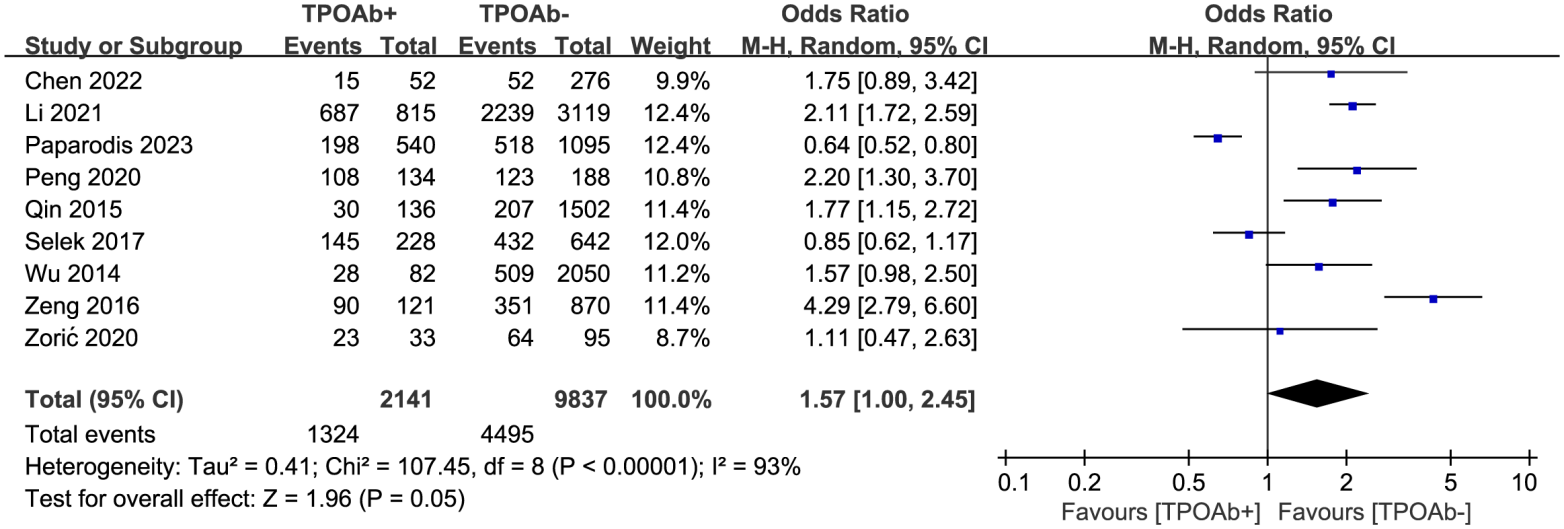
TPOAb positivity and prevalence of DTC.

To explore the association between TPOAb positivity and DTC prevalence, nine studies (39, 41–45, 48–50) were included. DTC prevalence was 62% among 2141 TPOAb+ patients and 46% among 9837 TPOAb– patients. However, no significant association between different TPOAb levels and the risk of developing DTC (OR=1.57 [95% CI: 1.00–2.45], p=0.05) was shown in the forest plot (**Figure 2**). As the p-value of 0.05 falls exactly on the critical threshold, repeated calculations were conducted using Stata 14, and a value of p=0.049 was ascertained. Egger’s regression model did not indicate any publication bias (p=0.45), but a high level of heterogeneity was observed.

### TPOAb positivity and tumor size

**Figure 3:**
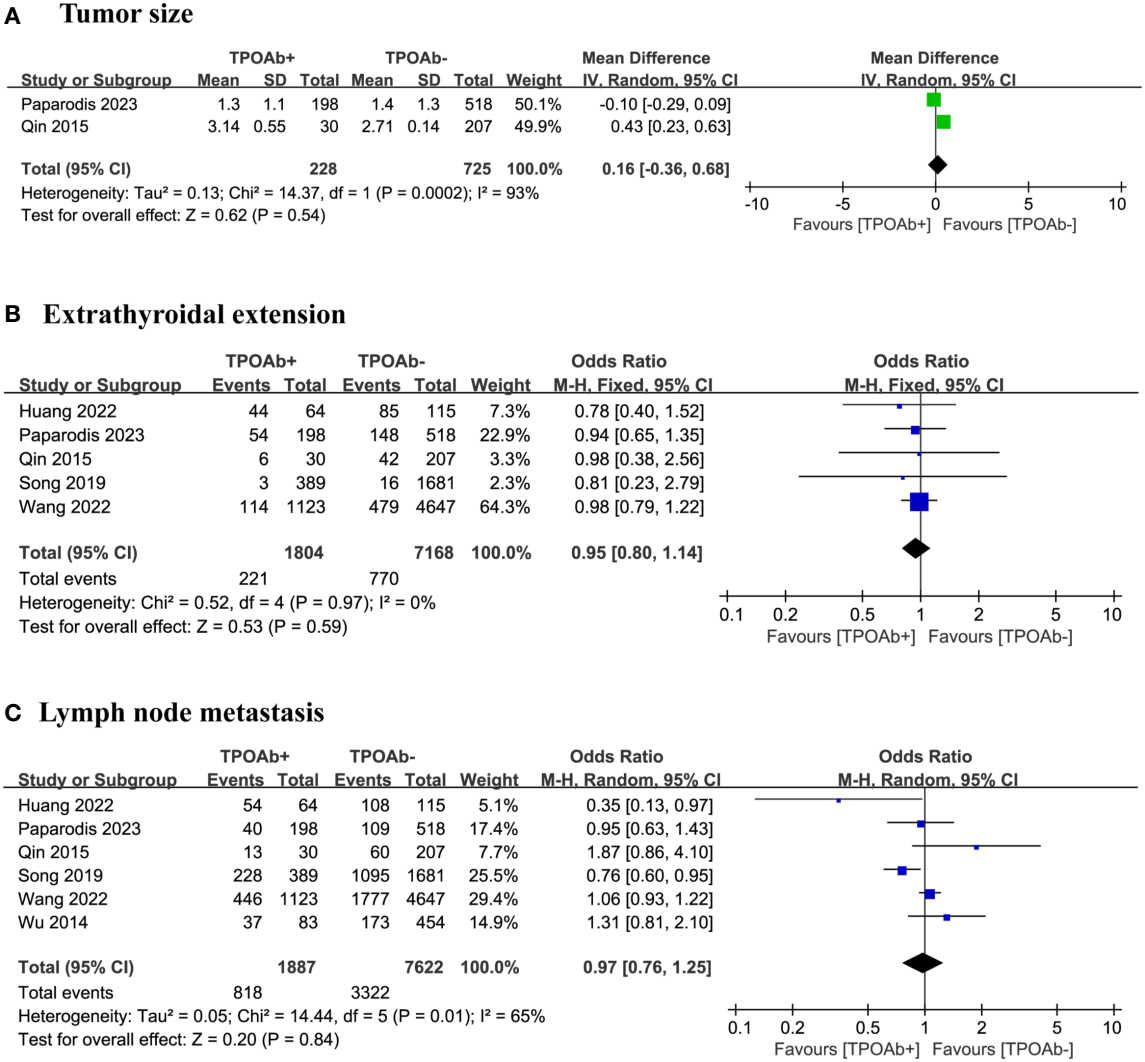
TPOAb positivity and risk of **(A)** tumor size, **(B)** extrathyroidal extension and **(C)** lymph node metastasis.

In our meta-analysis, two studies (42, 44) were selected to compare tumor size with various TPOAb levels. The results did not reveal a significant relationship between different TPOAb levels and tumor size (p=0.54; **Figure 3A**). However, the number of studies was insufficient for assessing publication bias, and a high level of heterogeneity was observed.

### TPOAb positivity and extrathyroidal extension

Our analysis focused on five studies (40, 42, 44, 46, 47) to determine whether the risk of extrathyroidal extension differs among DTC patients with various TPOAb levels. Among the 1804 TPOAb+ and 7168 TPOAb– patients, little difference in the risk of extrathyroidal extension was found, 12% and 11%, respectively. The forest plot did not indicate a significant association between different TPOAb levels and the risk of extrathyroidal extension (OR=0.95 [95% CI: 0.80–1.14], p=0.59; **Figure 3B**). Egger’s regression model showed no publication bias (p=0.19), and a low level of heterogeneity was observed.

### TPOAb positivity and lymph node metastasis

We included six studies (40, 42, 44, 46–48) to explore the relationship between TPOAb positivity in DTC patients and the risk of lymph node metastasis. Among the 1887 TPOAb+ and 7662 TPOAb– patients, little difference in the risk of lymph node metastasis was found, 43% and 44%, respectively. The forest plot did not show a significant association between different TPOAb levels and the risk of lymph node metastasis (OR=0.97 [95%CI 0.76-1.25] p=0.84; **Figure 3C**). Egger’s regression model indicated no publication bias (p=0.82), and a moderate level of heterogeneity was observed.

### TPOAb positivity and tumor multifocality

**Figure 4:**
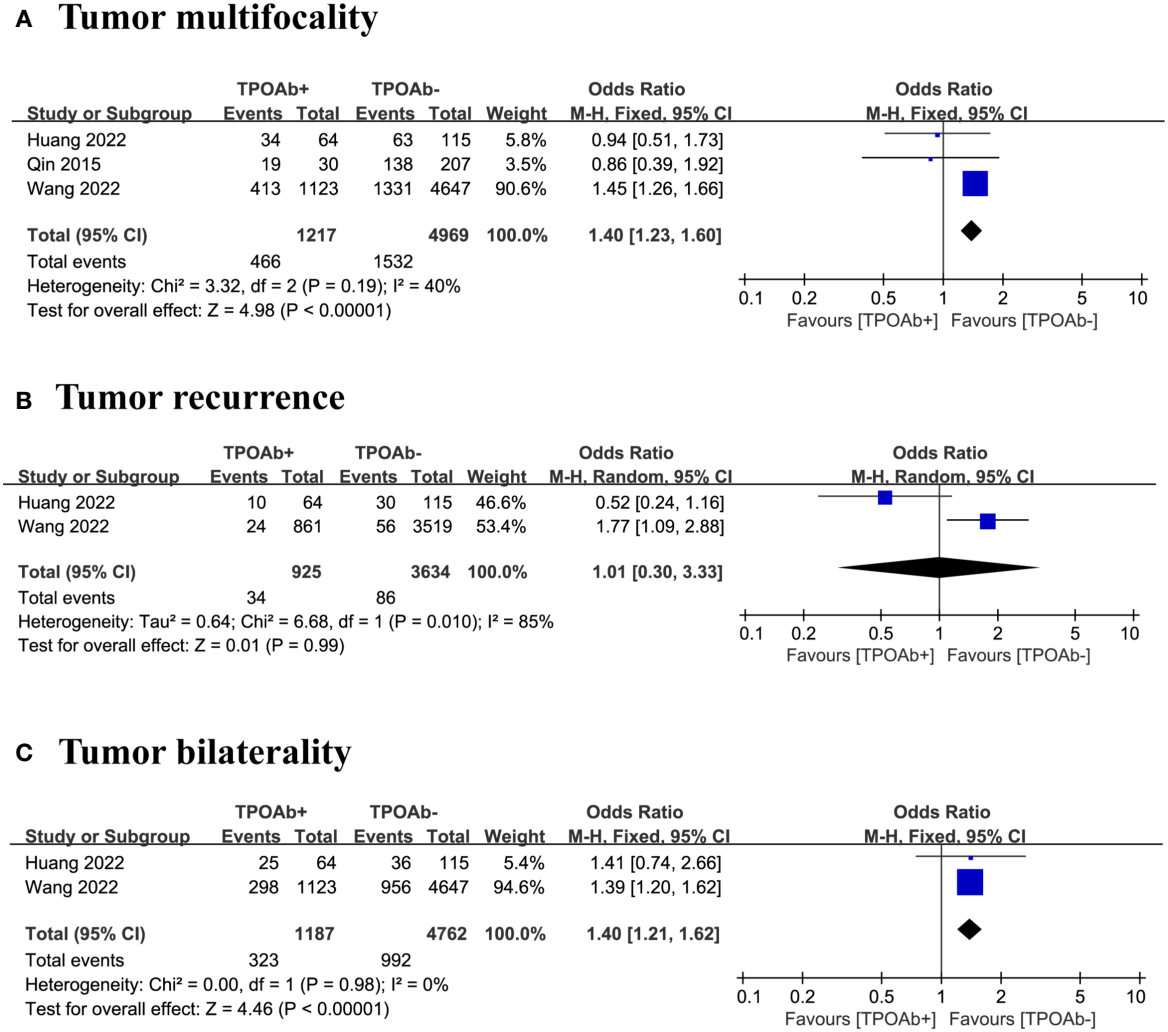
TPOAb positivity and risk of **(A)** tumor multifocality, **(B)** tumor recurrence and **(C)** tumor bilaterality.

We included three studies (40, 44, 47) to examine the association between TPOAb positivity and the risk of tumor multifocality in DTC patients. Multifocal tumors occurred in 38% of the 1217 TPOAb+ patients compared with 31% of the 4969 TPOAb– patients. The forest plot indicate the risk of tumor multifocality in TPOAb+ patients was significantly higher than that in TPOAb– patients (OR=1.40 [95% CI: 1.23–1.60], p<0.00001; **Figure 4A**). Egger’s regression model indicated no publication bias (p=0.05), and a low level of heterogeneity was observed.

### TPOAb positivity and tumor recurrence

Our analysis focused on two studies (40, 47) to evaluate the association between TPOAb positivity and the risk of tumor recurrence in DTC patients. Tumor recurrence occurred in 4% of the 925 TPOAb+ patients compared with 2% of the 3634 TPOAb– patients. The forest plot did not reveal a significant relationship between different TPOAb levels and the risk of tumor recurrence (OR=1.01 [95% CI: 0.30–3.33], p=0.99; **Figure 4B**). However, the number of studies was insufficient for assessing publication bias, and a high level of heterogeneity was observed.

### TPOAb positivity and tumor bilaterality

We included three studies (40, 47) to evaluate the association between TPOAb positivity and the risk of tumor bilaterality in DTC patients. Of the 1187 TPOAb+ patients, 27% had bilateral tumors compared with 21% of the 4762 TPOAb– patients. The risk of tumor bilaterality in TPOAb+ patients was significantly higher than that in TPOAb– patients (OR=1.40 [95% CI: 1.21–1.62], p<0.00001; **Figure 4C**). However, the number of studies is insufficient for assessing publication bias, and a low level of heterogeneity was observed.

### Sensitivity analysis

We conducted a sensitivity analysis to assess the stability of the results, systematically excluding each article and performing a meta-analysis on the remaining literature. Except for the high sensitivity of the results for TPOAb positivity and DTC prevalence and its association with the risk of bilateral and multifocal tumors of DTC, the remaining findings exhibited no substantial alterations, suggesting some degree of result instability.

## Discussion

Our meta-analysis included 12 original studies encompassing 20,330 patients from six countries for investigating associations between TPOAb and DTC prevalence, along with various prognostic factors. Our findings reveal that TPOAb+ individuals were at a higher risk of developing DTC than TPOAb– individuals (p=0.049). Furthermore, TPOAb+ DTC patients were more likely than TPOAb– DTC patients to present with multifocal and bilateral tumors, and this difference was statistically significant. However, no significant difference in tumor size, recurrence rate, risk of extrathyroidal extension, and lymph node metastasis was observed between TPOAb+ and TPOAb– DTC patients.

We observed an association between TPOAb and DTC prevalence, but the results may be subject to controversy. Upon analysis using Review Manager, we obtained a calculated p-value of 0.05, while recalculation using Stata 14 yielded a p-value of 0.049, which was statistically significant. This discrepancy might be because Review Manager rounded the p-value, and ultimately, the p-value was determined to be 0.049. Nevertheless, both the heterogeneity and sensitivity of this result were relatively high. Several factors may account for this observed heterogeneity. First, variations in the cutoff values for defining TPOAb positivity were evident, ranging from a minimum of >5.61 IU/mL to a maximum of >100 IU/mL. Second, differences existed in the methods and instruments used for TPOAb determination. Third, differences in the study populations across diverse regions may have contributed to heterogeneity. Lastly, the presence or absence of AIT in TPOAb+ patients could have influenced the results. The available data were not sufficient for a comprehensive subgroup analysis. Furthermore, the observed sensitivity issues were likely related to ethnic and regional differences. In the nine included articles, three studies contributing to the elevated sensitivity were from joint research conducted between the United States and Greece, Serbia, and Turkey, while the remaining six studies were conducted in China. Located in Asia, China differs geographically from the other four countries primarily situated in Europe and North America. As DTC prevalence varies across regions, geographic factors might play a pivotal role (51, 52). Although the p-value was <0.05, this does not inherently guarantee result stability or high reliability. Further validation through additional independent studies is essential to establish the reliability of the results.

We initially observed that TPOAb+ DTC patients are more likely to exhibit bilateral and multifocal tumors. However, Wang’s study (47) with a considerably higher weight share in both results had a significant influence. Upon exclusion of the Wang’s study, we found no significant association between TPOAb and DTC multifocality (OR=0.91 [95% CI: 0.56–1.48], p=0.70) and bilaterality (OR=1.41 [95% CI: 0.74–2.66], p=0.29). Consequently, no conclusive significant association was established in this study, emphasizing the need for further exploration of the association of TPOAb positivity with DTC multifocality and bilaterality. Moreover, we found the TPOAb+ and TPOAb– DTC patients exhibited no significant differences in tumor size, recurrence rate, risk of extrathyroidal extension, and lymph node metastasis. Nevertheless, several studies have reported that TgAb+ DTC patients face a higher risk of lymph node metastasis and recurrence (53–57).

Some studies have demonstrated that persistent or recurrent thyroid cancer can induce the production of TgAb, so the time of TPOAb detection is particularly important (58, 59). Other studies have indicated an association between positive TPOAb results 7-10 years before diagnosis and thyroid cancer. However, most of the original studies included in this analysis conducted TPOAb testing prior to surgery without specifying the exact duration of TPOAb positivity before this period, potentially impacting the credibility of the results (60). Further large prospective studies are necessary for validation. Additionally, if patients were identified with thyroid cancer due to testing positive for TPOAb and subsequently included in the selection process, it could have led to an increased rate of DTC diagnosis in TPOAb+ patients, potentially introducing selection bias. Moreover, because patients with autoimmune thyroid diseases tend to be more vigilant about their thyroid function, they may detect small subclinical tumors during ongoing medical surveillance. In contrast, patients without related diseases might not undergo continuous surveillance, potentially leading to the undiagnosed status of some subclinical tumors. This discrepancy could also introduce bias into the results.

Given that TPOAb is a vital diagnostic marker of HT, considering the influence of AIT when examining the association between TPOAb and DTC is necessary. HT is known to cause substantial immune cell infiltration in the thyroid gland (61), and similar immune cell infiltration is observed around DTC (62, 63). The potential interplay between the two and its effect on DTC progression and prognosis pose intriguing questions. HT appears to be linked to an increased risk of thyroid cancer (64). When it coexists with thyroid cancer, it seems to further elevate the likelihood of thyroid cancer development (64–66). However, it may confer a protective effect against lymph node metastasis, extrathyroidal extension, and distant metastasis (64, 67, 68). Additionally, some studies have shown that immune cell infiltration surrounding PTC resulting from HT is positively correlated with a lower recurrence rate, higher overall survival, and reduced risk of extrathyroidal extension and distant metastasis (69, 70). Moreover, some studies have found that HT is associated with smaller tumor size, lower rate of aggressive PTC variants and lower risk, and DTC patients with HT have a higher clinical remission rate and longer recurrence-free survival (71, 72). However, the accuracy of “non-HT status” as a negative prognostic marker is poor, and it cannot improve the specificity of predicting prognosis. In one meta-analysis, TgAb was distinguished from HT, and TgAb+ patients with HT exhibited larger tumor sizes and a higher risk of extrathyroidal extension, tumor multifocality, lymph node metastasis, and cancer persistence than those without HT (29). Therefore, although HT can promote the development of DTC, it seems to be a protective factor for the prognosis of DTC. However, if we consider the single effect of TgAb positivity without considering HT, it seems to become a risk factor. The studies included in our meta-analysis did not differentiate TPOAb from HT, making it challenging to determine whether our conclusions might have been confounded by HT. Further research is warranted to reach a more definitive conclusion.

## Conclusions

Our analysis indicates that TPOAb positivity may be associated with an increased prevalence of DTC. However, TPOAb does not serve as a robust prognostic factor for TPOAb+ DTC patients. Our meta-analysis is based on a limited number of included studies, and we anticipate that future research will provide additional large-scale research data to further inform our understanding.

## Data availability statement

The original contributions presented in the study are included in the article/**Supplementary Material**, further inquiries can be directed to the corresponding author.

## Author contributions

HZ: Writing – original draft. LT: Writing – review & editing. XW: Writing – review & editing. XS: Writing – review & editing.
