# Supplementary material for: The relationship between thyroid peroxidase antibody and differentiated thyroid cancer: a systematic review and meta-analysis
Source: Front Endocrinol (Lausanne). 2024 Feb 27;15:1349041. doi: 10.3389/fendo.2024.1349041 (PMC10927769; doi:10.3389/fendo.2024.1349041)
Supplement: Supplementary file 1 [file DataSheet_1.docx]

Supplementary Table 1. Search strategy

Pubmed

(((((differentiated thyroid cancer) OR (differentiated thyroid carcinoma)) OR ((((((Thyroid cancer, Hurthle cell[Supplementary Concept]) OR (Hurthle Cell Thyroid Neoplasia)) OR (Follicular thyroid cancer, Hurthle cell type)) OR (Hurthle cell carcinoma of the thyroid)) OR (Thyroid carcinoma, Hurthle cell)) OR (Thyroid cancer, follicular, Hurthle cell type))) OR ((((((((((Adenocarcinoma, Follicular[MeSH Terms]) OR (Adenocarcinomas, Follicular)) OR (Follicular Adenocarcinoma)) OR (Follicular Adenocarcinomas)) OR (Thyroid Carcinoma, Follicular)) OR (Follicular Thyroid Carcinoma)) OR (Carcinoma, Follicular Thyroid)) OR (Carcinomas, Follicular Thyroid)) OR (Follicular Thyroid Carcinomas)) OR (Thyroid Carcinomas, Follicular))) OR ((((((((((((((((((((Thyroid Cancer, Papillary[MeSH Terms]) OR (Cancer, Papillary Thyroid)) OR (Cancers, Papillary Thyroid)) OR (Papillary Thyroid Cancer)) OR (Papillary Thyroid Cancers)) OR (Thyroid Cancers, Papillary)) OR (Thyroid Carcinoma, Papillary)) OR (Carcinoma, Papillary Thyroid)) OR (Carcinomas, Papillary Thyroid)) OR (Papillary Thyroid Carcinomas)) OR (Thyroid Carcinomas, Papillary)) OR (Papillary Carcinoma Of Thyroid)) OR (Papillary Thyroid Carcinoma)) OR (Familial Nonmedullary Thyroid Cancer)) OR (Nonmedullary Thyroid Carcinoma)) OR (Carcinoma, Nonmedullary Thyroid)) OR (Carcinomas, Nonmedullary Thyroid)) OR (Nonmedullary Thyroid Carcinomas)) OR (Thyroid Carcinoma, Nonmedullary)) OR (Thyroid Carcinomas, Nonmedullary))) AND (((thyroid peroxidase antibody) OR (TPOAB)) OR ((((thyroid microsomal antibodies[Supplementary Concept]) OR (thyroid peroxidase activity-inhibiting immunoglobulins)) OR (TM antibodies)) OR (TMII))) 130

Web of Science

| # | Searches | Results |
| --- | --- | --- |
| 1 | (((((TS=(thyroid microsomal antibodies)) OR TS=(thyroid peroxidase activity-inhibiting immunoglobulins)) OR TS=(TM antibodies)) OR TS=(TMII)) OR TS=(thyroid peroxidase antibody)) OR TS=(TPOAb) | 16508 |
| 2 | ((((((((((((((((((((TS=(Thyroid Cancer, Papillary)) OR TS=(Cancer, Papillary Thyroid)) OR TS=(Cancers, Papillary Thyroid)) OR TS=(Papillary Thyroid Cancer)) OR TS=(Papillary Thyroid Cancers)) OR TS=(Thyroid Cancers, Papillary)) OR TS=(Thyroid Carcinoma, Papillary)) OR TS=(Carcinoma, Papillary Thyroid)) OR TS=(Carcinomas, Papillary Thyroid)) OR TS=(Papillary Thyroid Carcinomas)) OR TS=(Thyroid Carcinomas, Papillary)) OR TS=(Papillary Carcinoma Of Thyroid)) OR TS=(Papillary Thyroid Carcinoma)) OR TS=(Familial Nonmedullary Thyroid Cancer)) OR TS=(Nonmedullary Thyroid Carcinoma)) OR TS=(Carcinoma, Nonmedullary Thyroid)) OR TS=(Carcinomas, Nonmedullary Thyroid)) OR TS=(Nonmedullary Thyroid Carcinomas)) OR TS=(Thyroid Carcinoma, Nonmedullary)) OR TS=(Thyroid Carcinomas, Nonmedullary)) | 36912 |
| 3 | (((((((((TS=(Adenocarcinoma, Follicular)) OR TS=(Adenocarcinomas, Follicular)) OR TS=(Follicular Adenocarcinoma)) OR TS=(Follicular Adenocarcinomas)) OR TS=(Thyroid Carcinoma, Follicular)) OR TS=(Follicular Thyroid Carcinoma)) OR TS=(Carcinoma, Follicular Thyroid)) OR TS=(Carcinomas, Follicular Thyroid)) OR TS=(Follicular Thyroid Carcinomas)) OR TS=(Thyroid Carcinomas, Follicular) | 16149 |
| 4 | (((((TS=(Thyroid cancer, Hurthle cell)) OR TS=(Hurthle Cell Thyroid Neoplasia)) OR TS=(Follicular thyroid cancer, Hurthle cell type)) OR TS=(Hurthle cell carcinoma of the thyroid)) OR TS=(Thyroid carcinoma, Hurthle cell)) OR TS=(Thyroid cancer, follicular, Hurthle cell type) | 1962 |
| 5 | (TS=(differentiated thyroid cancer)) OR TS=(differentiated thyroid carcinoma) | 18253 |
| 6 | #2 OR #3 OR #4 OR #5 | 52074 |
| 7 | #1 AND #6 | 371 |

Embase

| # | Searches | Results |
| --- | --- | --- |
| 1 | 'thyroid peroxidase antibody'/exp OR 'thyroid peroxidase autoantibody' OR 'thyroid peroxidase antibody' | 7633 |
| 2 | 'thyroid papillary carcinoma'/exp OR 'columnar cell variant of PTC' OR 'cribriform-morular variant of PTC' OR 'diffuse sclerosing variant of PTC' OR 'follicular variant of PTC' OR 'hobnail variant of PTC' OR 'oncocytic variant of PTC' OR 'papillary cancer of the thyroid' OR 'papillary cancer of thyroid' OR 'papillary carcinoma of the thyroid' OR 'papillary carcinoma of thyroid' OR 'papillary microcarcinoma of the thyroid' OR 'papillary microcarcinoma of thyroid' OR 'papillary microcarcinomas of the thyroid' OR 'papillary thyroid cancer' OR 'papillary thyroid carcinoma' OR 'papillary thyroid microcarcinoma' OR 'tall cell variant of PTC' OR 'thyroid cancer, papillary' OR 'thyroid gland papillary carcinoma' OR 'thyroid papilla carcinoma' OR 'thyroid papillary cancer' OR 'thyroid papillary microcarcinoma' OR 'thyroidal papillary carcinoma' OR 'Warthin-like variant of PTC' OR 'thyroid papillary carcinoma' | 29356 |
| 3 | 'thyroid follicular carcinoma'/exp OR 'angioinvasive ftc' OR 'carcinoma, thyroid follicle' OR 'carcinoma, thyroidal gland follicle' OR 'encapsulated angioinvasive carcinoma, thyroid gland' OR 'encapsulated angioinvasive ftc' OR 'follicle carcinoma, thyreoideal gland' OR 'follicle carcinoma, thyroid gland' OR 'follicular cancer of the thyroid' OR 'follicular cancer of the thyroid gland' OR 'follicular carcinoma of the thyroid' OR 'follicular carcinoma of the thyroid gland' OR 'follicular carcinoma of thyroid' OR 'follicular carcinoma of thyroid gland' OR 'follicular thyroid cancer' OR 'follicular thyroid carcinoma' OR 'follicular thyroid gland carcinoma' OR 'minimally invasive ftc' OR 'thyroid encapsulated angioinvasive carcinoma' OR 'thyroid follicle carcinoma' OR 'thyroid gland encapsulated angioinvasive carcinoma' OR 'thyroid gland follicle carcinoma' OR 'thyroid gland follicular carcinoma' OR 'thyroidal encapsulated angioinvasive carcinoma' OR 'thyroidal follicle carcinoma' OR 'thyroidal follicular carcinoma' OR 'thyroidal gland encapsulated angioinvasive carcinoma' OR 'thyroidal gland follicle carcinoma' OR 'thyroidal gland follicular carcinoma' OR 'thyroideal encapsulated angioinvasive carcinoma' OR 'thyroideal follicle carcinoma' OR 'thyroideal follicular carcinoma' OR 'thyroideal gland encapsulated angioinvasive carcinoma' OR 'thyroideal gland follicle carcinoma' OR 'thyroideal gland follicular carcinoma' OR 'well-differentiated follicular carcinoma' OR 'widely invasive ftc' OR 'thyroid follicular carcinoma' | 8271 |
| 4 | 'Hurthle cell carcinoma'/exp OR 'Huerthle cell carcinoma' OR 'Huerthle cell thyroid cancer' OR 'Huerthle cell thyroid carcinoma' OR 'Hurthle cell adenocarcinoma' OR 'Hurthle cell cancer' OR 'Hurthle cell thyroid cancer' OR 'Hurthle cell thyroid carcinoma' OR 'oncocytic (Hurthle cell) carcinoma' OR 'oxyphilic carcinoma of the thyroid' OR 'oxyphilic subtype of thyroid cancer' OR 'oxyphilic thyroid carcinoma' OR 'Hurthle cell carcinoma' | 934 |
| 5 | 'differentiated thyroid cancer'/exp OR 'differentiated carcinoma of the thyroid' OR 'differentiated thyroid cancers' OR 'differentiated thyroid carcinoma' OR 'differentiated thyroid carcinomas' OR 'DTC (differentiated thyroid cancer)' OR 'WDTC (well differentiated thyroid cancer)' OR 'well differentiated thyroid cancer' OR 'well differentiated thyroid cancers' OR 'well differentiated thyroid carcinoma' OR 'well differentiated thyroid carcinomas' OR 'well-differentiated cancer of the thyroid' OR 'well-differentiated carcinoma of the thyroid' OR 'differentiated thyroid cancer' | 15200 |
| 6 | #2 OR #3 OR #4 OR #5 | 44392 |
| 7 | #1 AND #6 | 366 |

Cochrane Library

| # | Searches | Results |
| --- | --- | --- |
| 1 | thyroid microsomal antibodies or thyroid peroxidase activity-inhibiting immunoglobulins or TM antibodies or TMII or thyroid peroxidase antibody or TPOAB | 1063 |
| 2 | MeSH descriptor: [Thyroid Cancer, Papillary] explode all trees | 73 |
| 3 | (Familial Nonmedullary Thyroid Cancer or Thyroid Carcinoma, Nonmedullary or Nonmedullary Thyroid Carcinoma or Carcinomas, Nonmedullary Thyroid or Carcinoma, Nonmedullary Thyroid or Thyroid Carcinomas, Nonmedullary or Nonmedullary Thyroid Carcinomas or Carcinoma, Papillary Thyroid or Papillary Thyroid Cancers or Carcinomas, Papillary Thyroid or Papillary Thyroid Cancer or Papillary Thyroid Carcinoma or Cancers, Papillary Thyroid or Thyroid Carcinoma, Papillary or Papillary Carcinoma Of Thyroid or Cancer, Papillary Thyroid or Thyroid Carcinomas, Papillary or Papillary Thyroid Carcinomas or Thyroid Cancers, Papillary) | 497 |
| 4 | #2 OR #3 | 497 |
| 5 | MeSH descriptor: [Adenocarcinoma, Follicular] explode all trees | 52 |
| 6 | (Carcinomas, Follicular Thyroid or Follicular Thyroid Carcinomas or Thyroid Carcinomas, Follicular or Follicular Adenocarcinoma or Thyroid Carcinoma, Follicular or Carcinoma, Follicular Thyroid or Adenocarcinomas, Follicular or Follicular Adenocarcinomas or Follicular Thyroid Carcinoma) | 161 |
| 7 | #5 OR #6 | 161 |
| 8 | (Thyroid cancer, Hurthle cell or Hurthle Cell Thyroid Neoplasia or Follicular thyroid cancer, Hurthle cell type or Hurthle cell carcinoma of the thyroid or Thyroid carcinoma, Hurthle cell or Thyroid cancer, follicular, Hurthle cell type) | 30 |
| 9 | (differentiated thyroid cancer or differentiated thyroid carcinoma) | 733 |
| 10 | #4 OR #7 OR #8 OR #9 | 1109 |
| 11 | #1 AND #10 | 11 |
